# On Sanguineous Tumours of the Head in New-Born Children

**Published:** 1836-01

**Authors:** 


Art. IV.—*Von der blutigen Kopfgeschwulst der Neugeborenen.* (*On Sanguineous Tumours of the Head in New-born Children.*) By Karl Unger, Professor in the Königsberg University, &c. *Beiträge zur Klinik der Chirurgie. Leipzig*, 1833.

*Observations on Sanguineous Tumours of the Head which form spontaneously, sometimes denominated Cephalæmatoma, and Abscessus Capitis Sanguineus Neonatorum.* By E. Geddings, M.D. Professor of Anatomy and Physiology in the University of Maryland. (*North American Archives. July*, 1835.)

The existence of sanguineous tumours on the heads of new-born children is extremely common; but we know, from our own observation, that practitioners of moderate experience, and at least average observation, are frequently perplexed as to their nature, and the mode of treatment they require, in consequence of the very brief and imperfect descriptions that are given of the disease by writers,* and judging from the printed reports of lectures, by English teachers of midwifery. The French and German writers have paid more attention to this subject, and it well merits the consideration of the practitioner. We proceed, therefore, to give an abstract of the papers before us, both of which present a good practical account of the disease. Dr. Geddings describes the disease as a soft fluctuating tumour, containing blood, and mostly situated on the right parietal bone. These tumours are frequently mistaken for hernia of the brain, and by many writers have been described under the name of "hernia cerebri," a very different and much more alarming disease. The tumour is generally discovered at birth, but sometimes not for several days after; there is seldom more than one, but sometimes two or more exist, either in communication with each other, or perfectly isolated. They are mostly small, slightly prominent, smooth and soft upon the surface, and circumscribed by well defined borders, which, in the majority of cases, when examined with attention, are found hard and elevated, conveying the sensation of a hard bony edge, surrounding the outline of the tumour, and an intermediate excavation, as if the outer table of the skull had been removed. Sometimes they are more diffused and flattened, and less clearly circumscribed. Zeller and Naegele state, that in

* Burns, for example, our latest and best authority upon such subjects, gives but nine lines to the description of these tumours. Midwifery, eighth Edit. p. 611.

a few instances, the hard elevated boundary is either entirely absent, or confined to a portion of the circumference of the tumour. In all cases, when they are properly developed, a distinct fluctuation can be perceived, and by making pressure upon the part, the point of the finger can be brought to bear upon the solid bottom of the cavity. The skin is seldom discoloured, but presents a pale, shining appearance, and generally the part is so little sensible, that considerable pressure gives but slight pain. In some cases the fluctuation is not so distinct: the tumour is soft and spongy to the feel, and its nature is then difficult to detect. The size of the tumour varies: it may either remain stationary or progressively increase. In some rare cases a pulsation can be felt in the part, shewing that the tumour communicates with one or more arteries. The tumour, when opened, is always found to contain blood, which varies, however, in appearance, according to the extent and duration of the disease; the condition of the soft parts and the bone; and the intimacy of the connexion between the cavity of the tumour and the small adjacent blood-vessels. The longer the blood is confined, the more deteriorated it becomes; and if the disease be allowed to continue until the bony structures are involved, it may acquire an offensive odour. Suppuration, too, may take place, and convert the tumour into a purulent abscess. Great difference of opinion exists as to the precise situation of the fluid, and its relations with the component parts of the scalp and the subjacent bone. Dr. Geddings, adopting very nearly the arrangement of Velpeau,* divides sanguineous abscesses, or tumours of the head, into five orders, according to the depth at which the fluid is situated.

1. *Between the skin and the aponeurosis of the scalp.* In this situation the fluid is superficial, and least apt to become diffused. This is the most simple and the most frequent variety of the disease, and is free from danger. It may easily be confounded with others, which form in consequence of the pressure sustained by the head of the child during labour, or of injury inflicted by the forceps. As the union between the integuments of the scalp and the aponeurosis of the occipito-frontalis muscle is very compact and unyielding, when the blood accumulates in this situation, it diffuses itself with great difficulty. Such tumours are, therefore, generally small, rounded, prominent, convex upon the surface, and surrounded by a well defined, hard, elevated border, as in tumours produced by blows upon the head. As the fluid is not in contact with the bone, these superficial tumours rarely give rise to serious consequences, and may be dispersed by proper discutient applications, or readily cured by evacuating their contents.

2. *Between the aponeurosis of the occipito-frontalis and the pericranium.* The characters of this variety differ from the former. The proximity of the disease to the surface of the bone renders it more hazardous than when it is superficial. As the blood accumulates beneath the aponeurosis, it is resisted by it, in its tendency to protrude towards the surface; but, in the direction of the circumference, it dissects up the loose cellular tissue which unites the aponeurosis to the pericranium, and diffuses itself extensively between the two structures, and over the surface of the head, until its further progress is arrested by a solid barrier,

* Traité des Accouchemens, t. ii. p. 589.

the indurated border already adverted to, which is set up by the process of adhesive inflammation. Hence such tumours are flatter, more expanded, less prominent than the preceding, and often less clearly defined; for there is not always the hard prominent boundary, or, if it is present, it is confined to a limited portion of the circumference of the swelling, so that the fluid diffuses itself, until it spreads out irregularly over a large extent of the head. The interposition of the aponeurosis renders the fluctuation more obscure, and if the fluid be allowed to remain too long, the pericranium and bone may be injured: caries and exfoliation of the latter may occur, and the case may thus end in the death of the patient. But, as in this variety the pericranium is interposed between the fluid and the bone, the latter does not generally become much affected, except when the tumour has existed a long time, and an early opening is not made to evacuate the blood.

3. *Between the pericranium and the bone.* This is a rare variety of the disease: it is more serious than either of the preceding, from the immediate contact of the fluid with the denuded bone, and the great danger in consequence of necrosis or caries, especially when the fluid is not early absorbed, or when a timely puncture is not made to give exit to it. The injury the bone suffers, varies. Sometimes it is slight, consisting merely of superficial caries and exfoliation; or the outer table becomes necrosed, and is thrown off. The entire thickness of the skull may be detached and the dura mater exposed, but this is a very rare occurrence. Fatal cases of this kind, however, are recorded. It must be difficult, during the life of the patient, to distinguish this variety from the preceding, and fortunately this is not of much consequence as far as the first is concerned: but it may be important to discriminate between this form of bloody tumour, and those which form in the diploë, because it would be unsafe to puncture some of the latter.

4. *Sanguineous tumours of the diploë of the skull.* Dr. Geddings confesses it may be questionable how far bloody tumours in this situation agree in their fundamental characters with those before described. Michaelis* affirms that the outer table of the cranium is always destroyed by necrosis or caries, and that the accumulation of blood beneath the pericranium is the consequence. Chilius remarks, that though apparently situated in the diploë of the bone, these tumours really occupy the space between it and the pericranium, and that what was mistaken for the outer table was the pericranium in a state of ossification. Dr. G. admits the possibility of such a mistake, yet there is incontestible evidence that tumours of the kind do sometimes form in the diploë of the bone, and give rise to all the consequences attributed to them. But the question recurs,—Are they identical in their nature with those which form in the tissues of the scalp, and between them and the bone? Both tend to destroy the bone, both may exist at birth, or form shortly after. Both are filled with blood: but when those of the scalp are laid open, they do not bleed after their contents are discharged, while some of those of the diploë continue to pour out blood so copiously as to endanger or destroy the patient. The organization of the diploë explains, to a certain extent,

* Dr. G. refers to Loder's Journal, which it may be inconvenient for the English reader to consult. An abstract of Michaelis's opinion is given by Underwood. Vide ninth Edit. by Dr. Marshall Hall, p. 485.—REV.

this striking peculiarity. The reticulated structure between the bones is not only abundantly supplied with arteries, but it is likewise traversed by large veins, which freely anastomose, and form an intricate venous plexus within the bony texture. These vessels communicate freely, by numerous small branches through the two tables of the skull, with the vessels of the scalp, and with those of the meninges, and also with the great sinuses of the latter. Hence it is probable that most of the bloody tumours which form in the diploë, originate in a varicose condition of these veins; that in proportion as the tumour increases in size, the tables of the bones are forced asunder or destroyed; that they are, in short, aneurismal tumours, and therefore pour out blood to a dangerous degree when opened. The great facility with which these veins become distended, the extent to which they may dilate, and the encroachment they must make, when thus affected, upon the tables of the skull, and, after these have been destroyed, upon the scalp, the meninges, and the brain, explain the general phenomena of the disease, and its fatal tendency. Sometimes, when the walls of the cranium are completely perforated, and the tumour attains a considerable size, it assumes all the appearances of congenital hernia cerebri, and may, like that disease, be forced into the cranium by pressure.

5. *Between the cranium and dura mater.* Dr. G. is of opinion that erectile tumours, analogous to those already described, may arise in the space between the cranium and dura mater, and he thinks it probable that many of the cases which have been described as examples of fungus of the dura mater, and congenital hernia cerebri, were instances of such erectile tumours.

The symptoms of constitutional disturbance produced by these tumours will evidently vary, with their duration, size, and relation to the adjacent parts. Dr. G. is inclined to doubt that these tumours are caused by pressure upon the head of the child in the act of labour, but the difficulty of arriving at any satisfactory conclusion upon this point, amidst many conflicting opinions, is admitted.

It is sometimes difficult to distinguish sanguineous tumours from congenital encephalocele, or hernia of the brain, and it is crteain that the two diseases have been very commonly confounded with each other. Both are soft and spongy to the feel, and impart to the touch the sensation of a depression or defect of the corresponding portion of the cranium. Both are often attended with pulsations which are synchronous with those of the heart, and in both there is a hard, well defined border: thus far they agree; but distinctions exist between them. When pressure is made on a hernia cerebri the tumour can be forced down upon the brain through the opening in the cranium, but when the pressure is made, vertigo, dimness of vision, loss of consciousness, or even convulsions will arise. Sanguineous tumours will not disappear on pressure, nor does pressure on them usually give rise to any such symptoms. If, too, pressure is made upon a hernia cerebri, no resistance is felt by the finger, while in sanguineous tumours it is soon arrested by the rough surface of the bone. Although pulsation is a character common to both affections, it is feeble in sanguineous tumours, and is only present during the earliest periods of their existence. In hernia cerebri the pulsation is stronger, and never entirely disappears.

*Treatment.* Experience proves that the great majority of these sanguineous tumours will disappear spontaneously, or that they may be dispersed by proper treatment: puncture or incision is therefore rarely necessary. Even if the tumour is of a very large size, it may be sometimes dispersed by any of the ordinary evaporating lotions. Leeches are sometimes required. Mercurial frictions, as recommended by Dieffenbach, can seldom or never be admissible. Pressure upon the tumour is rarely useful or safe. It is difficult to determine how far the treatment can be confided to these means, as in some cases the disease has been allowed to continue for weeks or months without mischief; while in others the cranium has been affected with necrosis or caries in a short time. Much injury, then, may sometimes result from relying too long upon discutient applications, and neglecting to let out the fluid by puncture. In order not to incur any risk it will be advisable, after the ordinary local remedies have been applied in vain for eight, ten, or fourteen days, to puncture the tumour, and evacuate its contents: upon this point there is no difference of opinion. Various modes have been practised of opening the tumour; e. g. by a seton, caustic, free incisions, and puncture. The last is unquestionably the best, the puncture being of sufficient size to admit the free exit of the fluid. When the contents of the tumour have been evacuated, a probe should be introduced through the orifice, to ascertain the state of the bone. If the bone is healthy, a small piece of lint should be introduced into the puncture, to prevent it from closing until the fluid ceases to accumulate within. But if the bone be denuded, rough and carious, the tumour should be freely laid open, and the wound dressed with digestive ointment. It generally happens that the fluid accumulates after its first evacuation; the tent of lint should then be withdrawn, to allow the fluid to escape, and if adhesions have formed to such an extent as to prevent it from flowing, they may be broken up with a probe. After the puncture, the part will require different treatment, according to its condition. It has been proposed, when the parts are very indolent, to throw stimulating injections into the cavity, to excite adhesive inflammation. Cases may occur to demand this practice, but it must be employed with great caution. The prognosis is generally favourable, except when the disease has been allowed, by too long delay, to destroy the cranial bones. Most writers represent the opening of these tumours to be perfectly free from danger: such an opinion is erroneous, for Goelis, Braun, Steuart, &c., have related instances which terminated fatally after being punctured, although the bone was not diseased. We can do but little for those tumours which sometimes form within the diploë, or within the cranium: fatal hemorrhage would probably ensue if they were opened, but if the inner table of the skull is entire, and if we could positively distinguish them from other tumours, an opening might be made, and if alarming hemorrhage should follow, it might be commanded by a graduated compress. The enlarged vessels would then become obliterated, and the cavity would heal up by granulation.

Such is the substance of the excellent practical lesson given by Dr. Geddings upon this hitherto neglected disease by English writers. It is an excellent summary of all that is known upon the subject, and the merely English reader would seek in vain for the information it conveys, in any work to which he could have access.

Professor Unger's paper is very brief, and may be briefly dismissed. It consists principally of the relation of four cases, which are detailed with even more than ordinary German minuteness. He does not enter into a general description of the different varieties of these tumours, as he presumes that every German reader is well acquainted with them. He considers the diagnosis of the different species difficult, and often impossible. In one case which he saw, exfoliation of the external table of the skull took place, but healthy granulations formed, and the child recovered. Two other cases terminated fatally, from total destruction of the bones and inflammation of the brain, as it was presumed, for examination post mortem was not permitted. Experience has taught M. Unger that the situation of the fluid in the tumour may generally be determined by attention to the following points. The more diffused and the less fluctuating the tumour, the greater the probability of the fluid being under the aponeurosis of the occipito-frontalis muscle. On the contrary, the prominence of the swelling, with well defined and evident hard edges, leads to the conclusion that the fluid is under the pericranium, for the yielding aponeurosis offers less opposition to the diffusion of the blood than the closely adherent pericranium. He places but little confidence in the pulsation of the tumour, which is regarded by many writers as a pathognomonic sign of the presence of fluid under the pericranium, for he has only remarked it during the process of suppuration. He agrees with Dr. Geddings, that disease of the bone is by no means a constant accompaniment of the tumour, and that when it is present, it is very generally an accidental complication. The cause of the swelling he attributes, with Naegele, to an original disease of the veins, and his opinion is confirmed by his having seen the bloody tumour of the head complicated with nævus.*

We have seen many cases of these sanguineous tumours on the heads of new-born children, and upon some few points we differ from Dr. Geddings. We believe, with Velpeau,† that pressure on the head, during labour, is the ordinary cause of these tumours. We have generally seen them after difficult and protracted labours, in which the head of the child has borne long and severe pressure. That they are seen, occasionally, after easy and natural labours, we do not deny, but presuming, as we do, that M. Unger is correct in his idea of the pathological character of the disease, it is easy to suppose that a sanguineous tumour might form on the head, or any other part of the body, when the veins were diseased, from even the slight pressure of a very favourable labour. We do not remember a single case in which there was a "distinct fluctuation" in the tumour, at any period of its progress. The sensation imparted to our touch, was that of a doughy, spongy substance: and it has seldom happened that

* In an interesting paper, "von den Muttermälern des Kopfes," in the same work, M. Unger states it as his belief that the nævus, termed by us aneurism by anastomosis, always depends upon a preternatural weakness and expansion of the parietes of the veins of the part. He does not admit that the arterial branches are affected. Upon this subject there is still some doubt; but we have arrived at the same conclusion as M. Unger, not merely from observing the appearance of the disease, but after having carefully injected and dissected several specimens of bloody nævus, the "tumeurs variqueuses" of Boyer, or the "tumeurs érectiles" of Dupuytren. REV.

† Accouchemens, t. ii. p. 595. Second Edition.

we could, by any pressure we could justifiably apply, bring the point of the finger to bear upon the solid bottom of the cavity. We will not deny that the common varieties of these tumours may lead to destruction of the bones of the cranium, like the very rare form in which the effused blood is contained between the tables of the bones, but we believe such a circumstance to be so very uncommon as very rarely to require our puncturing the tumour. Time and patience, and evaporating lotions, which are perhaps employed more to keep up the appearance of doing something, than because they are absolutely necessary, is all that is required in the great majority of such cases. But while we impress this general rule, we would caution the young practitioner especially, to profit by the remarks of Dr. Geddings, and not forget the exceptions to it.

